# Sequence-Based Genotyping for Marker Discovery and Co-Dominant Scoring in Germplasm and Populations

**DOI:** 10.1371/journal.pone.0037565

**Published:** 2012-05-25

**Authors:** Hoa T. Truong, A. Marcos Ramos, Feyruz Yalcin, Marjo de Ruiter, Hein J. A. van der Poel, Koen H. J. Huvenaars, René C. J. Hogers, Leonora. J. G. van Enckevort, Antoine Janssen, Nathalie J. van Orsouw, Michiel J. T. van Eijk

**Affiliations:** Keygene N.V., Wageningen, The Netherlands; Nanjing Agricultural University, China

## Abstract

Conventional marker-based genotyping platforms are widely available, but not without their limitations. In this context, we developed Sequence-Based Genotyping (SBG), a technology for simultaneous marker discovery and co-dominant scoring, using next-generation sequencing. SBG offers users several advantages including a generic sample preparation method, a highly robust genome complexity reduction strategy to facilitate *de novo* marker discovery across entire genomes, and a uniform bioinformatics workflow strategy to achieve genotyping goals tailored to individual species, regardless of the availability of a reference sequence. The most distinguishing features of this technology are the ability to genotype any population structure, regardless whether parental data is included, and the ability to co-dominantly score SNP markers segregating in populations. To demonstrate the capabilities of SBG, we performed marker discovery and genotyping in *Arabidopsis thaliana* and lettuce, two plant species of diverse genetic complexity and backgrounds. Initially we obtained 1,409 SNPs for arabidopsis, and 5,583 SNPs for lettuce. Further filtering of the SNP dataset produced over 1,000 high quality SNP markers for each species. We obtained a genotyping rate of 201.2 genotypes/SNP and 58.3 genotypes/SNP for arabidopsis (n = 222 samples) and lettuce (n = 87 samples), respectively. Linkage mapping using these SNPs resulted in stable map configurations. We have therefore shown that the SBG approach presented provides users with the utmost flexibility in garnering high quality markers that can be directly used for genotyping and downstream applications. Until advances and costs will allow for routine whole-genome sequencing of populations, we expect that sequence-based genotyping technologies such as SBG will be essential for genotyping of model and non-model genomes alike.

## Introduction

Marker assisted selection (MAS) is used to significantly accelerate the plant breeding process. In MAS, molecular markers such as single nucleotide polymorphisms (SNPs) and simple sequence repeats (SSRs) are used to indirectly select for genetic determinant(s) of a trait of interest. Commercially important crop traits include abiotic stress tolerance, disease resistance, high yields, and improved nutritional qualities [1], [2]. MAS can offer advantages such as screening plants for a desired trait at very early growth stages, recurrent selection of desirable alleles at each cycle of crossing and breeding, and concurrent selection of multiple traits [3]. Beyond MAS, molecular markers are useful in studies of genetic variation, linkage mapping, population structure analysis, genome-wide association studies, and map-based gene isolation [4], [5], [6], [7], [8], [9].

Although the number of molecular markers has rapidly expanded with the development of high-throughput marker discovery and genotyping technologies, in some crop plants the number of markers remains surprisingly low [10]. In part this is a result of limitations specific to individual crops, such as those with polyploid or highly repetitive genomes, but technical and economic challenges also prohibit the identification of large numbers of molecular markers.

Next-generation sequencing (NGS) technologies are providing researchers with the unprecedented means to unravel the underlying sequence variation associated with heritable traits. The costs associated with sequencing complete plant genomes is one factor that still limits its' routine use in SNP discovery, especially when a reference genome is unavailable. Despite this, massively parallel sequencing, with a sequencing capacity from millions, to billions of bases per run, will significantly revolutionize the way in which SNP discovery and genotyping is achieved in the future [11].

Coupling genomic reduction strategies to NGS may further reduce the costs of detecting a large number of novel SNPs in a high-throughput manner. This requires however that genome complexity reduction is performed in a reproducible manner, in all samples to be sequenced in a specific experiment, but also over multiple experiments. Traditionally, methods such as the AFLP**®** technique [12] have proven to reduce genome complexity in a flexible and highly reproducible way. In AFLP, genome complexity reduction is achieved concurrently in a large number of individuals by simply varying the choice and number of restriction enzymes, as well as amplifying the resultant fragments with primers containing selective bases. The complexity reduction features of AFLP were successfully exploited by van Orsouw et al. for the discovery of high quality SNPs in maize by NGS [13].

There are currently several approaches that combine marker discovery and genotyping with the express aim to provide high quality markers in a single, synchronous step. These include sequencing of reduced representation libraries [14], restriction-site-associated DNA sequencing (RAD-seq) [15], multiplexed shotgun sequencing [16] and genotyping-by-sequencing (GBS) [17], [18]. Essentially, all of the aforementioned technologies comprise common key steps in their processes. At the core of each is the utilization of restriction enzyme(s) to facilitate genome complexity reduction amongst individuals or populations, and provide fixed starting points for sequencing. The resultant restriction fragments are further selected or reduced by various means, and the final set of fragments is sequenced by NGS. SNPs found between the sequenced fragments can directly be used as markers for genotyping [19].

In the present work, we describe Sequence-Based Genotyping (SBG). SBG incorporates the high-throughput capacity of NGS platforms, and the proven, reproducible and robust genome complexity reduction capabilities of AFLP, to score random SNP markers across an entire genome. Using SBG, genome-wide SNP discovery and genotyping of large populations can be attained in a single experiment, without the need for prior knowledge of a reference genome sequence. Depending upon the user's needs, this method allows for the customization of the type of complexity reduction required, the optimal number of samples to be analyzed, as well as the desired number of SNPs. Additionally, SBG markers and genotypes can be directly used for downstream applications, which in turn can bring added value to the user. Here we present applications of SBG in arabidopsis and lettuce populations, two plant species of diverse genetic complexity and backgrounds. We demonstrate that SBG is applicable to a wide range of species using a generic sample preparation process, and standardized bioinformatics analysis workflows for germplasm and parent-based genotyping.

## Materials and Methods

### DNA Samples

Total genomic DNA was isolated from leaf material using a modified CTAB procedure [20] from the following arabidopsis and lettuce populations:

#### Arabidopis

The *Arabidopsis thaliana* ecotype Columbia and the homozygous insertional mutant WiscDsLox353E12 (N852397, NASC, University of Nottingham, United Kingdom, http://arabidopsis.info/) were crossed to *A. thaliana* ecotype Landsberg. F1 plants were backcrossed to arabidopsis ecotype Landsberg resulting in two backcross (BC1) populations, one wild-type population (Col×Ler)×Ler and one mutant population (Col mutant×Ler)×Ler. The sampled population consisted of a total of 220 offspring plants resulting from the two aforementioned BC1 populations and the parental lines (n = 222 samples).

#### Lettuce

The sampled population consisted of 85 lettuce (*Lactuva sativa*) cv. Salinas 88× cv. La Brillante, generation eight recombinant inbred lines (RILs) and the parental lines (n = 87 samples) [21].

### Sequencing Sample Preparation

**Figure 1:**
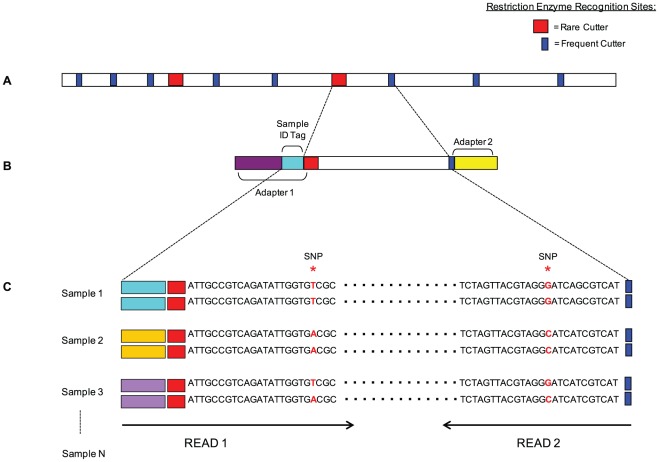
Overview of SBG. (A) The sequencing complexity of genomic DNA is reduced using a combination of rare and frequent cutting enzymes. (B) Sequencing adapters containing sample identification tags are ligated to the restriction fragments to construct SBG libraries. SBG libraries are amplified and sequenced using Illumina sequencing platforms. Only read 1 will be sequenced for single-end sequencing, while both read 1 and read 2 will be sequenced for paired-end sequencing. (C) SNPs are mined between the samples and simultaneously genotyped using the SBG bioinformatics analysis workflow.

Arabidopsis libraries were constructed for Illumina single-end sequencing whilst lettuce libraries were constructed for Illumina paired-end sequencing as follows (Figure 1):

#### Arabidopsis

In brief, 100–500 ng total genomic DNA was digested using 5 units *Eco*RI and 5 units *Mse*I for at least 1 hour at 37**°**C. Following digestion, the mixture was heated at 85**°**C for 10 minutes. Adapter ligation was then performed using a universal P7 *Mse*I adapter (top oligo: 5′-CAAGCAGAAGACGGCATACGAG-3′-; bottom oligo: 5′-TACTCGTATGCCGTCTTCTGCTTG-3′-NH_2_) and a sample-specific tagged *Eco*RI P5 adapter (top oligo: 5′-AATGATACGGCGACCACCGAGATCTACACTCTTTCCCTACACGACGCTCTTCCGATCTxxxxxC-3′; bottom oligo: 5′-AATTGxxxxxAGATCGGAAGAGCGTCGT-3′-NH_2_; xxxxx = sample identification tag) for 3 hours at 37**°**C. Sample-specific *Eco*RI P5 adapters contained a unique 5-nt sample identification tag adjacent to the *Eco*RI restriction site overhang for identification of individual samples, and were designed such that each sample identification tag differed by at least two bases from all other tags. A complete list of all sample identification tags used is shown in the supplementary materials (Table S1). PCR was performed in a total reaction volume of 20 µl containing 5 µl of 10-fold diluted restriction-ligation mixture, 5 ng Illumina P5 primer (5′-AATGATACGGCGACCACCG-3′), 30 ng Illumina P7 primer (5′-CAAGCAGAAGACGGCATACGA-3′), 0.2 mM dNTPs, 0.4 U AmpliTaq® (Applied Biosystems) and 1× AmpliTaq® buffer. PCR was performed with a cycle profile that consisted of 2 minutes at 72°C, followed by 50 cycles of 30 seconds at 94°C, 60 seconds at 58°C, and 2 minutes at 72°C. Reactions were held at 4°C until ready for use. Next, sets of 32 PCR amplified samples were pooled (5 µl each) to make 7 libraries and these were purified using the MinElute PCR Purification Kit (Qiagen). Single-end sequencing (76 nt) was performed using 7 lanes of the Illumina Genome Analyzer II (1 library per lane). Clusters for each library were generated on a GAIIx flow cell v2 using a Cluster Kit v5, according to manufacturer's instructions. Following the completion of the run, image analyses, error estimation and base calling were performed using the Illumina Pipeline (SCS 2.5/RTA 1.5.35.0) to generate primary data.

#### Lettuce

A two-step digestion was performed whereby 100–500 ng total genomic DNA was first digested with 5 units *Taq*I for 1 hour at 65**°**C. This was immediately followed by digestion with 5 units *Pst*I and 5 units *Mse*I for 1 hour at 37**°**C. The use of a third enzyme, in this case *Mse*I, allowed for additional genomic complexity reduction. Following digestion, the mixture was heated at 85**°**C for 10 minutes. Adapter ligation was then performed using a modified Illumina Paired-end *Taq*I P7 adapter (top oligo: 5′-CAAGCAGAAGACGGCATACGAGATCGGTCTCGGCATTCCTGCTGAACCGCTCTTCCGATCTG-3′; bottom oligo: 5′-CGCAGATCGGAAGAGCGGTTCAGCAGGAA-3′-NH_2_), modified Illumina Paired-end *Pst*I P5 adapter (top oligo: 5′-AATGATACGGCGACCACCGAGATCTACACTCTTTCCCTACACGACGCTCTTCCGATCTxxxxxATGCA-3′; bottom oligo: 5′-TxxxxxAGATCGGAAGAGCGTCGT-3′-NH_2_; xxxxx = sample identification tag) and an AFLP *Mse*I adapter (top oligo: 5′-GATGAGTCCTGAG-3′; bottom oligo: 5′-TACTCAGGACTCAT-3′), for 3 hours at 37**°**C. Paired-end *Pst*I P5 adapters contained a unique 5-nt sample identification tag adjacent to the *Pst*I restriction site overhang for identification of individual samples. As with arabidopsis, the sample identification tags differed by at least two bases. The full list of sample identification tags are shown in the supplementary materials (Table S1). Excess adapters from each restriction-ligation were removed using the Agencourt AMPure XP System (Beckman Coulter Genomics) for DNA purification and cleanup. Next, PCR was performed in a total reaction volume of 20 µl containing 5 µl of 10-fold diluted restriction-ligation mixture, 5 ng Illumina P5 primer (5′-AATGATACGGCGACCACCG-3′), 30 ng Illumina P7 primer (5′-CAAGCAGAAGACGGCATACGA-3′), 0.2 mM dNTPs, 0.4 U AmpliTaq® (Applied Biosystems), and 1× AmpliTaq® buffer. PCR was performed with a cycle profile consisting of 2 minutes at 72°C, followed by 50 cycles of 30 seconds at 94°C, 60 seconds at 58°C, and 2 minutes at 72°C. Reactions were held at 4°C until ready for use. Next, sets of 32 PCR amplified samples were pooled (5 µl each) to make 3 libraries. For each library, fragments were separated, sized and quantified using the Agilent High Sensitivity DNA Kit and loaded onto the Agilent 2100 Bioanalyser (Agilent Technologies) for evaluation. Paired-end sequencing (100 nt) was performed using 3 lanes of the Illumina HiSeq2000 (1 library per lane). Clusters for each library were generated on a HiSeq flow cell v3 using a TruSeq Paired-End Cluster Kit v3, according to manufacturer's instructions. Following the completion of the run, image analyses, error estimation and base calling were performed using the Illumina Pipeline (HCS 1.4.8/RTA v1.12.4.2) to generate primary data.

### Processing of the Illumina sequence data

**Figure 2:**
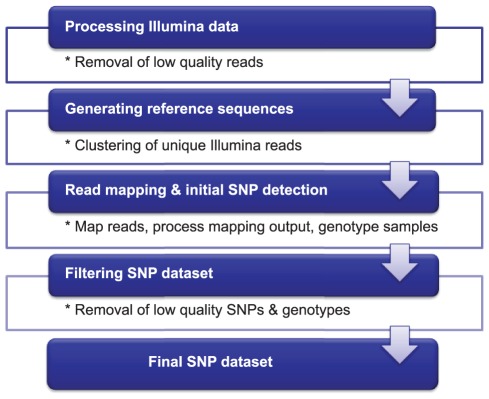
Bioinformatics analysis workflow for SBG. The Illumina data are first processed to remove low quality reads. The reference sequences are generated by clustering the unique reads present within the dataset. The reads are subsequently aligned to the reference sequences and variation called using the GATK Unified Genotyper. Lastly, the final set of SNPs and genotypes are generated by removing SNPs not meeting the threshold for percentage of missing data and expected genotypic frequencies.

The Illumina short read sequences were pre-processed by applying several filtering criteria (Figure 2). We removed from the dataset reads that did not contain the sample identification tag, and reads without one of the expected restriction enzyme motifs. In addition, we also discarded reads that contained homopolymeric stretches, had a positive hit against a chloroplast, mitochondria and repeat (for lettuce only) database, contained undetermined nucleotides (Ns), and displayed a low average quality score.

### Generation of the reference sequences

SEED [22] was used to create reference sequences to which the short reads were aligned. The datasets that were used as input for SEED were obtained by clustering all full length reads that passed the quality control, at 100% sequence identity. This clustering identified the number of unique reads present in the dataset, and how many times each unique read was observed. The number of reads used in clustering was determined after discarding unique reads found greater than 100,000 times, and less than 100 or 200 times, for lettuce and arabidopsis respectively. The lower thresholds were an approximation of the population size. The clusters obtained with the arabidopsis sequence data were subsequently mapped to the publicly available arabidopsis genome sequence, using BWA [23] as the mapping tool.

### Read mapping and variant calling

The short reads were aligned to the reference sequences using BWA and the mapping results were processed with Samtools [24]. Variation was called using the Unified Genotyper (Genome Analysis Tool Kit) [25]. Any nucleotide difference between the reads, or between the reads and the reference genome, was initially called as a variant. The resulting variant output was therefore large, and less reliable data points needed to be removed. We used three parameters generated by the Unified Genotyper as criteria to filter the variant output, namely genotype coverage, genotype quality, and SNP quality.

### SNP and genotype validation

In order to determine the impact of each of the thresholds on SNP and genotype validation rates, a set of 312 genotypes was selected from 8 SNPs. These SNPs were chosen on the basis of mapping the arabidopsis reads to the publicly available arabidopsis reference genome. The genotypes were selected at different coverage values, varying from 4× to 92×, and included approximately an equal number of homozygote and heterozygote genotypes as expected from the BC1 population that we used. Primers were designed for each of the 8 SNPs, and fragments amplified by PCR. Subsequently, Sanger sequencing was used to sequence the fragments and evaluate the genotypes. We considered that a Unified Genotyper call was correct when the genotype called was the same as the genotype determined by Sanger sequencing. Genotypes based on Sanger sequencing were independently called by two persons, and the results were matched afterwards. We discarded all Sanger genotypes that could not be called conclusively.

### SNP discovery and genotyping

Upon completion of the SNP and genotype validation step, a new dataset was generated by applying thresholds at which the SNP and genotype validation rates were maximized. The same thresholds were applied to both the arabidopsis and lettuce datasets. Moreover, we performed parent-based genotyping by determining the SNP positions where the parental samples were both present and fixed for alternate alleles, and genotyping the offspring samples that were detected at those positions. For each given SNP position, the genotypes were labeled as A or B, for the two types of homozygous genotypes, in accordance with the parental genotypes, and also H, for the heterozygous genotypes.

### Linkage mapping

All genetic maps for arabidopsis and lettuce were calculated using the CarteBlanche software package [26]. CarteBlanche is a genetic mapping software program which allows estimation of linkage groups, determination of the most likely map orders using various mapping algorithms, and varying visualization methods and statistics to judge map quality. *De novo* grouping/mapping of markers as well as anchor-grouping/mapping is supported. Recombination frequencies between markers were estimated using a likelihood-based approach. Recombination frequencies of 0.5 indicate that markers are unlinked. Thus, recombination frequencies greater than 0.4, are re-estimated through a shortest path algorithm using intermediate markers.

The first step in the mapping process was the assignment of markers to linkage groups, the genetic equivalent of chromosomes, based on observed recombination fractions. After formation of the initial linkage groups, the group contents were optimized by merging splitting groups as required, and by placing additional markers from the ungrouped set that can still be placed in one of the groups unambiguously.

The next step involved the estimation of pair-wise recombination frequencies, corresponding LOD scores, and genetic distances for all marker pairs in each linkage group. For each linkage group a genetic map was constructed using five different mapping algorithms. After completion, the best map was selected out of these preliminary results, based on minimal sum-of-adjacent-recombination-frequencies, and maximal sum-of-adjacent-LOD-scores. Finally, the genetic distances in this map were optimized.

The quality of the best map found was judged by plotting its marker order amongst those of the other maps, which shows the stability of the selected map. Frequent positioning of markers in one of the alternative maps in orders deviating from the best map, indicates that either insufficient information was present to obtain a definite solution, or a part of the locus segregation data conflicts. The quality of the map was also judged by evaluating genome configurations of the individuals of the population.

## Results

### Illumina sequencing

The total number of reads generated was approximately 177 M and 383 M in the arabidopsis and lettuce datasets, respectively. The average number of reads obtained per lane was 25.3 M in arabidopsis and 127.6 M in lettuce, which is in agreement with the throughput of the sequencing platform used to sequence each crop (Illumina Genome Analyzer-*II* in arabidopsis; HiSeq2000 in lettuce). After applying filtering criteria, the percentage of reads used in analyses varied from 57.7% (lettuce read 1) to 69.3% (arabidopsis), which is similar to what was observed in other studies [27].

The distribution of the number of reads per sample obtained in the arabidopsis and lettuce sequence datasets is indicated in Figures S1 and S2, respectively. For both crops, a normal distribution was observed for the number of reads that were assigned to each sample. This indicated that the SBG sample preparation procedure used resulted in an even distribution of reads between the various samples, even though some outliers were also identified.

All arabidopsis and lettuce sequences were submitted to the National Center for Biotechnology Information (NCBI) Short Read Archive (submission SRA052230).

### Reference sequences

The results of the strategy we used to generate sets of reference sequences are shown in Table 1. For all sequence datasets, only full length reads were used for generating reference sequences. The number of unique reads varied from approximately 3.5 M in arabidopsis, to 18.8 M in the lettuce read 2 dataset. In all sequence datasets, the vast majority of the unique reads were observed very few times. In arabidopsis, 75% of all unique reads were observed five times or less, while in lettuce this percentage was found to be approximately 89%. Consequently, after discarding the unique reads that were above or below the thresholds used, the number of reads used in clustering represented a small percentage of the total number of unique reads identified. The number of clusters identified varied directly with the number of reads used for clustering, and ranged from 13,321 clusters in arabidopsis to 168,759 in the lettuce read 2 dataset.

**Table 1:** Summary statistics for generating the reference sequences.

	Arabidopsis	Lettuce read 1	Lettuce read 2
**Number of filtered reads**	122,573,199	220,953,145	253,109,987
**Full length reads**	110,849,880	203,441,535	239,282,874
**% Full length reads**	90.4	92.1	94.5
**Unique reads**	3,500,146	9,869,623	18,849,951
**Reads used in clustering**	18,500	161,974	241,676
**Number of clusters**	13,321	107,661	168,759

The accuracy of the clustering strategy adopted was evaluated in arabidopsis. The clusters generated were mapped to the publicly available arabidopsis reference genome, and evaluated for their mapping position (Table S2). We defined the indicator of clustering quality as the percentage of clusters that could be mapped to a single location on the arabidopsis genome. A total of 14.4% of all clusters was either not mapped, or mapped to multiple locations on the arabidopsis genome. Hence, 85.6% of the clusters formed, totaling 11,408 clusters, were mapped to a unique position on the arabidopsis genome. A unique position was defined as a position on the sequence of the arabidopsis genome where a single cluster was mapped. The reason why we wanted to determine the number of unique positions was because it was possible for the same genome position to be covered by more than one cluster. A total of 11,248 single mapped clusters were detected, which represented 84.4% of the initial number of clusters formed with SEED. Finally, the number of clusters that mapped to the arabidopsis mitochondrial genome was also very low (0.2%), and no clusters were mapped to the arabidopsis chloroplast genome. We used these organelle genomes together with the arabidopsis genome to evaluate the number of clusters that would map to the mitochondrial or chloroplast genomes. Considering that we had removed the reads that had a positive hit against these organelle genomes during the initial processing of the Illumina sequencing data, we did not expect a significant number of clusters to map to these genomes. This was indeed the situation we observed.

### SNP and genotype validation

A set of 312 genotypes, were selected from a subset of 8 SNPs that were identified after mapping the arabidopsis reads to the reference genome. SNP and genotype data were generated using the Unified Genotyper as the tool to call variation. We then used Sanger sequencing to validate these SNPs and genotypes. A genotype was considered to be correct when the call by the Unified Genotyper and Sanger sequencing matched. A detailed description of the genotypes selected for validation is included in Table S3. The overall validation rate was high (96.5%). Only 11 of the 312 genotypes were incorrectly called when the genotype determined by Sanger sequencing was taken as the reference. The validation rate was higher for heterozygote genotypes, a class for which only two incorrect genotypes were detected. For this genotype class, high validation rates were observed when a minimum of two reads per allele was detected. This indicated that when compared to homozygote genotypes, the coverage threshold for heterozygote genotypes could be less stringent. The homozygote genotypes that were incorrectly called were shown to be heterozygote genotypes by Sanger sequencing. This result illustrates the risk that at low coverage values, one of the alleles of a heterozygote genotype is not sequenced, leading to a homozygote genotype being incorrectly called.

The validation rates obtained for the set of 312 genotypes subject to Sanger sequencing were further evaluated for the Unified Genotyper genotype quality parameter, which is the Phred-scaled confidence that the true genotype was called. Table S4 includes the validation rates obtained at different coverage (merging the data from both genotype classes) and genotype quality thresholds. The maximum validation rate observed was 99.2%, at the 7× and 8× coverage thresholds. The slight decrease observed at higher coverage values was explained by the fact that the number of incorrect genotypes remained constant beyond 7× coverage, but the total number of genotypes called decreased.

A minimum validation rate of 96.5% was observed when no threshold was placed on genotype quality, a parameter generated by the Unified Genotyper. The validation rate was highest at a minimum genotype quality of 20. This was the threshold at which 98.9% of the called genotypes were confirmed by Sanger sequencing. The results obtained for the validation of genotypes at different coverage and genotype quality thresholds, were the basis for establishing a default stringency level to consider a genotype to be valid or discarded. These thresholds were set at 7× coverage and 20 for genotype quality. An additional threshold for SNP quality, another parameter generated by the Unified Genotyper, was also included and set at 30. Hence, all genotypes we have presented in this study passed all these thresholds.

The initial variant output generated for each sequence dataset is summarized in Table 2. The number of variants identified varied from 6,799 in arabidopsis to 321,566 in the lettuce read 2 dataset, reflecting the amount of sequencing that was performed. It may also reflect the differences in the initial amount of genetic variation that existed in the populations we analyzed.

**Table 2:** Variant calling for the arabidopsis and lettuce sequence datasets.

Sequence dataset	Total number of variants	Number of contigs
**Arabidopsis**	6,799	3,360
**Lettuce read 1**	152,210	39,994
**Lettuce read 2**	321,566	60,279

The results of the parent-based genotyping performed in each sequence dataset are included in Table 3. Several filtering criteria such as coverage per genotype (7×), genotyping quality (20), and SNP quality (30), were applied to the SNP positions where both parents were present and fixed for alternate alleles. The number of SNPs identified with parent-based genotyping was smaller compared to the initial SNP output. This was a consequence of the requirements implemented for this SNP detection strategy. The number of SNPs identified varied from 1,409 in arabidopsis to 3,665 in the lettuce read 2 datasets. The frequency of genotypes B and H was very close to 50%, which was in accordance with the expectation for this type of BC1 population. Hence, the frequency for genotype A was very low (1.2%). This was another clear indication of high quality SNP discovery and genotyping in arabidopsis, since the A genotype should not be observed in this population, and its frequency could be regarded as an indicator of the genotyping error rate. In lettuce, the heterozygosity of the F8 RIL population was expected to be residual. Hence, a frequency of approximately 50% for each of the homozygote genotypes was anticipated. We observed that the genotypic frequency for the A (46.6%) and B (44.0%) genotypes was less than the expected frequency of 50%, and the H genotype (9.4%) was much higher than predicted.

**Table 3:** Parent-based SNP genotyping in the arabidopsis and lettuce sequence datasets.

	Arabidopsis	Lettuce read 1	Lettuce read 2	Lettuce all
**Number of SNPs**	1,409	1,918	3,665	5,583
**Total number of genotypes**	273,992	79,674	135,021	214,695
**Number genotypes/SNP**	194.5	41.5	36.8	38.5
**Number of A genotypes**	3,303	36,627	63,344	99,971
**Frequency genotype A**	1.2	46.0	46.9	46.6
**Number B genotypes**	139,628	35,787	58,734	94,521
**Frequency genotype B**	51.0	44.9	43.5	44.0
**Number H genotypes**	131,061	7260	12943	20,203
**Frequency genotype H**	47.8	9.1	9.6	9.4

### Additional filtering of SNPs and genotypes

The SNP dataset generated with parent-based genotyping was subjected to additional filtering steps to remove false-positives. For each identified SNP, thresholds were placed on the percentage of missing data and the frequency of each of the genotypes. SNPs that displayed an amount of missing genotypes above the threshold, or genotypic frequencies exceeding the thresholds, were removed from the dataset. We applied this additional filtering step to the SBG datasets generated with parent-based genotyping for both arabidopsis and lettuce (Table 4). For arabidopsis, we removed SNPs that displayed: i) more than 60% missing genotypes; ii) a frequency of more than 75% or less than 25% for the homozygote B and heterozygote genotypes; and iii) a frequency for the homozygote A genotype of more than 3%. The frequency of the latter genotype can be regarded as an indication of the error rate of the genotyping procedure. Hence, removing all SNPs with a frequency of the homozygote A genotype larger than 3% ensured an accuracy rate of at least 97%. The number of SNPs decreased from 1,409 to 1,245, which meant that 88.4% of the SNPs initially called in arabidopsis with parent-based genotyping were kept after application of the filtering criteria described above. In addition, the number of genotypes per SNP also increased from 194.5 to 201.2.

**Table 4:** Parent-based SNP genotyping in the arabidopsis and lettuce sequence datasets after removing SNPs displaying extreme genotypic frequencies and an excessive number of missing genotypes.

	Arabidopsis	Lettuce read 1	Lettuce read 2	Lettuce all
**Number of SNPs**	1,245	589	637	1,226
**Total number of genotypes**	250,517	34,991	36,440	71,431
**Number genotypes/SNP**	201.2	59.4	57.2	58.3
**Number of A genotypes**	2,035	16,626	17,407	34,033
**Frequency genotype A**	0.8	47.5	47.8	47.6
**Number B genotypes**	128,773	17,665	18,299	35,964
**Frequency genotype B**	51.4	50.5	50.2	50.3
**Number H genotypes**	119,709	700	734	1,434
**Frequency genotype H**	47.8	2.0	2.0	2.0

Although in arabidposis the additional filtering steps removed only a small percentage of the SNPs identified with parent-based genotyping, a much more pronounced effect was observed in lettuce. Similar rules were applied to filter the initial SNP output generated in lettuce parent-based genotyping. These rules included the removal of SNPs that displayed i) more than 60% missing genotypes; ii) a frequency of more than 75% or less than 25% for the homozygote genotypes; and iii) a frequency for the heterozygote genotype of more than 15%.

For lettuce, a higher percentage of SNPs were removed after application of these filtering criteria. In fact, 78% of the SNPs identified initially did not pass the filtering criteria applied, when data from both sequence datasets was considered. The number of SNPs removed was larger in the lettuce read 2 dataset, when compared with read 1. As a consequence, the differences observed between the two lettuce sequence datasets were also much smaller in the filtered SNP set. Finally, the average number of genotypes per SNP increased significantly (from 38.5 to 58.3) and the frequency of the heterozygote genotype decreased (from 9.4 to 2.0).

These results illustrate the gains that can be achieved in the quality of a SNP set after using filtering criteria based on expected genotypic frequencies, and desired percentage of missing genotypes. It should be emphasized that these results were obtained using only the data derived from parent-based genotyping. However, this strategy can be adopted for every SNP detected, regardless of the availability of parental information.

### Linkage Mapping using SBG markers

Both the arabidopsis and lettuce SNP datasets were subjected to linkage mapping using the CarteBlanche software package (Figure S3). For arabidopsis, the dataset converged into five distinct groups, corresponding to the five arabidopsis chromosomes. For the five groups, 150 map orders were generated. This resulted in a stable map configuration with more than 1200 mapped markers, and an average chromosome length of approximately 125 centimorgan (cM). Some groups were somewhat larger than what has been found in previous mapping studies for arabidopsis [28], [29]. This could, however, be an effect of the high-density dataset that we used for linkage mapping (on average more than 200 marker per chromosome). In such high-density datasets, there is more opportunity for single data points to inflate the map distances.

The lettuce SNP dataset proved to be more challenging, as the amount of missing data was relatively high for a mapping dataset. When we tried to map the complete dataset in a single pass, most markers were placed in a single group. This was likely due to the large amount of missing data. To circumvent this problem, a two-step approach was used. First a subset of 493 markers with the fewest missing data (up to 30%) was used to create the groups. This resulted in the formation of 27 groups of variable size, in which 481 of the 493 markers were present. A stable, high quality map was generated using the aforementioned subset of markers, based on 150 map orders. Using these groups as anchors, the remaining markers were then assigned to the group with the best fit, based on the recombination fraction. Using this type of approach, 632 of 733 markers with missing data in the range of 30–60%, could be unambiguously assigned to one of the groups.

For the 27 lettuce marker groups, 150 map orders were once again determined, and the best obtained order was preserved. Stability analysis of the 150 map orders showed that in most cases a stable marker order could be obtained. Some unstable regions were also observed but in the majority of the cases, these corresponded to regions with many markers within a small cM interval. When markers are very similar or co-segregating, the order amongst these markers becomes less defined. The final lettuce linkage map consisted of 1113 markers mapped out of a total of 1226 markers, and spanned 947.7 cM.

## Discussion

Conventional marker-based genotyping technologies have several disadvantages that can readily be improved through the use of a sequence-based method. Namely, genotyping platforms like SNP arrays are large-scale operations that require a substantial investment to initially discover SNPs, and subsequently genotype a large number of individuals. Moreover these systems tend to be limiting in flexibility and scalability of fixed ordering volumes, fixed number of SNPs per assay, and/or a relatively long lag time in ordering and receiving. Others are not well suited as high-throughput assays for a large number of SNPs, often requiring the design of allele specific primers [30].

Sequence-based genotyping methods such as SBG, combine SNP discovery and genotyping in one single step. This makes SBG considerably time and cost-effective in comparison to conventional genotyping technologies. SBG is also a generic technology, with minimal amount of pre-experimental setup since there is no additional primer, adapter or assay design required. This ensures that the project turnaround time is low, and the user has tangible results in just a few days. Multiplexing of samples using tagged adapters further contributes to the high-throughput nature of this technology, allowing a large number of samples to be screened in a given experiment. Lastly, the user gains invaluable information about the type, the location, and the sequence context of each SNP marker since SBG is sequence-based. This information can immediately be incorporated into existing sequence-based frameworks such as Whole Genome Profiling (WGP™) [31], or used in downstream applications such as QTL mapping, MAS, genetic distance analyses, and genome-wide association studies.

SBG offers several competitive advantages over other sequence-based SNP genotyping technologies. In comparison to RAD-seq for example, we have refined our library preparation protocol such that we have limited the amount of sample handling steps involved, reduced the number of PCR and purification steps, and we do not utilize DNA size fractionation. These measures help to increase the efficiency and ease of library preparation. Furthermore, the genome complexity reduction strategy that we apply is anchored within the highly robust and reproducible complexity reduction capabilities of AFLP. Unlike other sequence-based technologies, we utilize a combination of at least two restriction enzymes, one rare cutting and another frequent cutting. This strategy not only allows us to effectively reduce genome complexity, but in doing so we create an even distribution of genomic fragments covering the length of a given genome. Depending on individual genome specificities, we are able to tailor complexity reduction using the specific properties of certain restriction enzymes. This feature of SBG is especially important in the case of complex genomes such as lettuce whereby a methylation sensitive enzyme was used to avoid highly repetitive portions of the genome. Our method is also suited for further complexity reduction using selective amplification, which we have recently demonstrated [32]. In addition, either single end, or paired-end sequencing can be employed for SBG. Paired-end sequencing may be of particular benefit because the cost per SNP will be reduced compared to the cost per SNP derived from single-end sequencing.

### Enzyme selection

Enzyme combinations for use in SBG were selected based upon the expected complexity of the genomes under study, and from our past AFLP experience from which the genome complexity reduction power of our current technology is derived. For arabidopsis, the *Eco*RI/*Mse*I combination was chosen with the expectation that it would provide the maximal output of genome-wide SNP markers for a genome of its size [33].

Although lettuce has yet to be sequenced in its entirety, it is believed that the lettuce genome is very repetitive. This adds to the need for an effective complexity reduction method prior to sequencing. The highly methylated, repetitive fraction of the lettuce genome could potentially be avoided with the use of a methylation sensitive enzyme such as *Pst*I. From previous studies, approximately 50% of *Pst*I sites in maize were expected to be methylated [34], which we assumed to be similar in lettuce. We also used a three restriction enzyme combination (*Pst*I, *TaqI* and *Mse*I) to further reduce the complexity in lettuce. We chose *Mse*I, a frequent cutting enzyme, to remove any fragments containing an *Mse*I restriction site (i.e. *P-M; M-P; T-M; M-T*; fragments). Effectively, this is equivalent to using selective nucleotides in AFLP for complexity reduction, without the need for additional amplification steps.

The choice of complexity reduction is highly dependent on the nature and size of the genome under study. The core AFLP-based complexity reduction that we have used is highly robust and reproducible in all genomes to be sequenced. This gives the user the freedom to choose the best combination of restriction enzymes to achieve their genotyping goals, in terms of SNP numbers and distribution of markers across the genome.

### Removal of adapter dimers

Adapter dimers can potentially pose a problem, as these small fragments tend to be preferentially sequenced. Adapter dimers were also noted in the original GBS method, and were resolved through a series of adapter titrations to empirically determine the correct ratio of adapters to sample DNA ends. In the course of development, we noticed the effect of adapter dimers when we used paired-end adapters to prepare the lettuce samples for sequencing. We were able to resolve the adapter dimer issue, and simultaneously size-select our library fragments, by employing a strategy that included a step in which all fragments below 200 bp were removed immediately following adapter ligation. Libraries were considered suitable only if adapter dimers were absent and all remaining fragments were greater than 200 bp.

### Genotyping strategy based on population structure

We have shown that SBG is capable of genotyping populations regardless of whether a reference genome is available. Through the uniform and streamlined bioinformatics analysis workflows we have developed, we have given the user the flexibility to screen germplasm populations or to perform parent-based genotyping, as well as performing co-dominant scoring of segregating SNP markers in a given population. We emphasize that genotyping of any population structure is possible with SBG, whereas most sequence-based genotyping studies have relied upon the inclusion of parental genotypes for the purposes of SNP discovery [18], [35], [36], [37]. Not surprisingly, what we have seen is that the inclusion of parental data, as is the case with parent-based genotyping, will enable refinement of the SNP set used for genotyping, thus ensuring that there are fewer false-positive SNP markers.

Lastly, we have demonstrated that we could use the SBG SNP markers to construct high quality *de novo* linkage maps for arabidopsis and lettuce. This is unlike the approach used recently by Poland *et al.* 2012 in which a wheat linkage map was developed such that existing genetic markers were used to anchor the GBS SNP markers to the respective wheat chromosome. Our SBG approach therefore gives the user the possibility to construct linkage maps without the need for pre-existing genetic map frameworks, which may not always be available for a given genome.

In the future, we expect that advances in NGS will increase the propensity for sequence-based genotyping. With SBG, we have targeted users that require genotyping information for populations that may or may not have a reference sequence available, and population structures that do not necessarily include parental data. The latter is one important distinguishing feature that sets SBG apart from all other sequence-based genotyping technologies. Therefore we conclude that SBG offers users the greatest flexibility in achieving their genotyping goals. Any future improvements to SBG can only positively contribute to this.

## Supporting Information

Figure S1 **Distribution of the number of reads per sample in the arabidopsis sequence dataset.**
(EPS)

Figure S2 **Distribution of the number of reads per sample in the lettuce sequence dataset.**
(EPS)

Figure S3 **Example of a typical linkage group for arabidopsis (A) and lettuce (B).**
(EPS)

Table S1 **Set of 96 sample identification tags used for SBG library construction.**
(DOC)

Table S2 **Results of mapping the clusters formed with SEED to the arabidopsis genome.**
(DOC)

Table S3 **Validation of arabidopsis SNP genotypes at different coverage thresholds.**
(DOC)

Table S4 **Impact of GATK Unified Genotyper thresholds on genotype validation rate.**
(DOC)
