# Supplementary material for: Sequence-Based Genotyping for Marker Discovery and Co-Dominant Scoring in Germplasm and Populations
Source: PLoS One. 2012 May 25;7(5):e37565. doi: 10.1371/journal.pone.0037565 (PMC3360789; doi:10.1371/journal.pone.0037565)
Supplement: Table S1 — Set of 96 sample identification tags used for SBG library construction. (DOC) [file pone.0037565.s004.doc]

**Table S1.** Set of 96 sample identification tags used for SBG library construction

| AACAC | AACCG | AACGT | AACTA | AAGGC | ACACT |
| --- | --- | --- | --- | --- | --- |
| ACTAT | ACTCC | ACTGA | ACTTG | AGGAC | AGGCG |
| CACTG | CAGAC | CAGCG | CAGGT | CAGTA | CATAG |
| CGCAG | CGCCA | CGCGC | CGCTT | CGGAA | CGGTC |
| CTTAC | CTTCG | CTTGT | GAACG | GAAGT | GAATA |
| GGTAC | GGTCG | GGTGT | GGTTA | GTACT | GTAGG |
| GTTAT | GTTCC | GTTGA | TAACT | TAAGG | TACAG |
| TATCG | TATGT | TATTA | TCAGA | TCCAC | TCCGT |
| ACAGG | ACCAG | ACCGC | ACCTT | ACGAA | ACGTC |
| AGGTA | ATAAC | ATACG | ATAGT | ATATA | ATTGC |
| CCAAC | CCACG | CCAGT | CCATA | CGACT | CGAGG |
| CGTAT | CGTCC | CGTGA | CGTTG | CTATG | CTGAG |
| GCCAT | GCGAC | GCGCG | GCGGT | GCGTA | GGAAT |
| GTCAG | GTCCA | GTCGC | GTCTT | GTGAA | GTGTC |
| TACCA | TACGC | TACTT | TAGAA | TAGTC | TATAC |
| TCCTA | TCGCA | TGAAC | TGACG | TGAGT | TGATA |
