# Supplementary material for: Sequence-Based Genotyping for Marker Discovery and Co-Dominant Scoring in Germplasm and Populations
Source: PLoS One. 2012 May 25;7(5):e37565. doi: 10.1371/journal.pone.0037565 (PMC3360789; doi:10.1371/journal.pone.0037565)
Supplement: Table S2 — Results of mapping the clusters formed with SEED to the arabidopsis genome. (DOC) [file pone.0037565.s005.doc]

**Table S2.** Results of mapping the clusters formed with SEED to the arabidopsis genome

| **Number of clusters (from SEED)** | **13,321** |
| --- | --- |
| **Not mapped** | 1,376 |
| **% Not mapped** | 10.3 |
| **Mapped to multiple locations** | 537 |
| **% Mapped to multiple locations** | 4.1 |
| **Mapped to unique location Arabidopsis genome** | 11,408 |
| **% Mapped to unique location** | 85.6 |
| **Arabidopsis genome positions with single mapped cluster** | 11,248 |
| **% Single mapped clusters** | 84.4 |
| **Mapped to mitochondrial genome** | 29 |
| **% Mapped to mitochondrial genome** | 0.2 |
