# Supplementary material for: Sequence-Based Genotyping for Marker Discovery and Co-Dominant Scoring in Germplasm and Populations
Source: PLoS One. 2012 May 25;7(5):e37565. doi: 10.1371/journal.pone.0037565 (PMC3360789; doi:10.1371/journal.pone.0037565)
Supplement: Table S3 — Validation of arabidopsis SNP genotypes at different coverage thresholds. (DOC) [file pone.0037565.s006.doc]

**Table S3.** Validation of arabidopsis SNP genotypes at different coverage thresholds

|  | **All genotypes** | | | **Homozygote genotypes** | | **Heterozygote genotypes** | |
| --- | --- | --- | --- | --- | --- | --- | --- |
| **Coverage** | **Total** | **Correct** | **Incorrect** | **Total** | **Incorrect** | **Total** | **Incorrect** |
| **4x** | 17 | 15 | 2 | 12 | 2 | 5 | 0 |
| **5x** | 16 | 13 | 3 | 9 | 2 | 7 | 1 |
| **6x** | 23 | 19 | 4 | 11 | 3 | 12 | 1 |
| **7x** | 19 | 19 | 0 | 10 | 0 | 9 | 0 |
| **8x** | 21 | 21 | 0 | 7 | 0 | 14 | 0 |
| **9x** | 15 | 15 | 0 | 7 | 0 | 8 | 0 |
| **10x** | 17 | 17 | 0 | 11 | 0 | 6 | 0 |
| **11x** | 12 | 12 | 0 | 6 | 0 | 6 | 0 |
| **12x** | 8 | 8 | 0 | 4 | 0 | 4 | 0 |
| **13x** | 12 | 11 | 1 | 9 | 1 | 3 | 0 |
| **14x** | 12 | 11 | 1 | 6 | 1 | 6 | 0 |
| **15x** | 6 | 6 | 0 | 4 | 0 | 2 | 0 |
| **>15x** | 134 | 134 | 0 | 62 | 0 | 72 | 0 |
| **TOTAL** | 312 | 301 | 11 | 158 | 9 | 154 | 2 |
