# Supplementary material for: Sequence-Based Genotyping for Marker Discovery and Co-Dominant Scoring in Germplasm and Populations
Source: PLoS One. 2012 May 25;7(5):e37565. doi: 10.1371/journal.pone.0037565 (PMC3360789; doi:10.1371/journal.pone.0037565)
Supplement: Table S4 — Impact of GATK Unified Genotyper thresholds on genotype validation rate. (DOC) [file pone.0037565.s007.doc]

**Table S4.** Impact of GATK Unified Genotyper thresholds on genotype validation rate

| **Coverage** | **Total number genotypes** | **Correct genotype calls** | **Incorrect genotype calls** | **Genotype validation rate (%)** |
| --- | --- | --- | --- | --- |
| **4x** | 312 | 301 | 11 | 96.5 |
| **5x** | 295 | 284 | 9 | 96.9 |
| **6x** | 279 | 273 | 6 | 97.8 |
| **7x** | 256 | 254 | 2 | 99.2 |
| **8x** | 237 | 235 | 2 | 99.2 |
| **9x** | 216 | 214 | 2 | 99.1 |
| **10x** | 201 | 199 | 2 | 99.0 |
| **>10x** | 184 | 182 | 2 | 98.9 |
|  | | | | |
| **Genotype quality** | **Total number genotypes** | **Correct genotype calls** | **Incorrect genotype calls** | **Genotype validation rate (%)** |
| **All** | 312 | 301 | 11 | 96.5 |
| **>10** | 310 | 300 | 10 | 96.8 |
| **>15** | 296 | 288 | 8 | 97.3 |
| **>20** | 275 | 274 | 3 | 98.9 |
| **>25** | 254 | 251 | 3 | 98.8 |
| **>30** | 245 | 242 | 3 | 98.8 |
